# Cost Effective Use of a Thiosulfinate-Enriched *Allium sativum* Extract in Combination with Chemotherapy in Colon Cancer

**DOI:** 10.3390/ijms21082766

**Published:** 2020-04-16

**Authors:** Jose Manuel Perez-Ortiz, Eva Maria Galan-Moya, Miguel Angel de la Cruz-Morcillo, Juan Francisco Rodriguez, Ignacio Gracia, Maria Teresa Garcia, Francisco Javier Redondo-Calvo

**Affiliations:** 1 Translational Research Unit, Hospital General Universitario de Ciudad Real, 13004 Ciudad Real, Spain; EvaMaria.Galan@uclm.es (E.M.G.-M.); sabesdemas@gmail.com (M.A.d.l.C.-M.); fjredondo@sescam.jccm.es (F.J.R.-C.); 2 Department of Medical Sciences, School of Medicine, Universidad de Castilla-La Mancha, 13071 Ciudad Real, Spain; 3 Translational Oncology Laboratory, Centro Regional de Investigaciones Biomédicas (CRIB), Universidad de Castilla-La Mancha, 02008 Albacete, Spain; 4 Department of Chemical Engineering, Institute of Chemical and Environmental Technology, Universidad de Castilla-La Mancha, 13071 Ciudad Real, Spain; Juan.RRomero@uclm.es (J.F.R.); Ignacio.Gracia@uclm.es (I.G.); teresa.garcia@uclm.es (M.T.G.); 5 Department of Anesthesiology, Hospital General Universitario de Ciudad Real, 13004 Ciudad Real, Spain

**Keywords:** thiosulfinate, garlic, allicin, HT-29, Caco-2, chemotherapy

## Abstract

In this work, we sought to investigate the effects of a thiosulfinate-enriched garlic extract, co-administered with 5-fluorouracil (5-FU) or oxaliplatin chemotherapy, on the viability of colon cancer cells (Caco-2 and HT-29). We also addressed the economic feasibility of a new combined treatment of this thiosulfinate-enriched garlic extract, with oxaliplatin that could reduce the dosage and costs of a monotherapy. The thiosulfinate-enriched garlic extract not only enhanced the impact of 5-FU and oxaliplatin (500 µM) in decreasing Caco-2 and HT-29 viability, but also showed a higher effect than standard 5-FU and oxaliplatin chemotherapy as anti-cancer agents. These results provided evidences for the combination of lyophilized garlic extract and 5-FU or oxaliplatin as a novel chemotherapy regimen in colon cancer cells that may also reduce the clinical therapy costs.

## 1. Introduction

Today, the use of natural sources instead of chemical derivatives in pharmaceutical industry is a development sector within the “Biorefinery” concept worldwide [1]. Structural diversity is a striking feature of natural products (NPs), accounting for their lasting importance in drug discovery [2,3,4]. In addition, most NPs show more favorable behavior compared to synthetic molecules, due to their biological properties. Moreover, they can be easily tailored, modified or blended for new drug targets [5]. 

One of the most cited NP for pharmacological applications during the last years is garlic (*Allium sativum*) and its derivatives. The therapeutic properties of garlic have been known, researched and used for more than 5000 years by different cultures, both for the treatment and prophylaxis of a great number of pathologies [6]. Among the properties that have been attributed to garlic throughout the history, it has been highlighted its antitumoral, immunomodulatory, antioxidant, bacteriostatic, anti-inflammatory, antiviral, antiparasitic and bactericidal capacities [7]. Therefore, garlic and its bioactive compounds are promising as functional foods or nutraceuticals for the prevention and treatment of different diseases [8].

In general, the therapeutic use of garlic has taken as reference the properties that have been attributed to allicin in most of the studies carried out [9]. In contrast, the instability of this compound has led to several researchers to focus on the properties of other compounds derived from allicin such as ajoene [10], which has demonstrated longer stability and more manageable presentations (U.S. Patent No. 5948821; U.S. Patent No. 5981602). Other groups have been involved in the synthetic preparation of allicin from alliinase and alliin, the precursor of allicin, whose in situ generation and application in tumor cells in vitro has demonstrated an antiproliferative effect (U.S. Patent No. 6689588). However, there are some studies that claim that synthetic allicin is more unstable than natural allicin [11], and that the natural alliinase from garlic chemically differs from that obtained from processed garlic, such as can be detected in natural garlic or even dehydrated garlic [12]. On the other hand, it has been demonstrated that isolated natural allicin generates a less relevant bacteriostatic effect than when it acts in conjunction with other garlic compounds, such as allyl substituted thiosulfinates [13,14], suggesting a synergistic effect of the compounds present in garlic.

Due to a generalized lack of chemical stability [15], standardization in production processes [16] and chemical characterization in clinical studies [17], research based on the use of garlic derivatives has led to ambiguous conclusions about the beneficial effects of these derivatives, thus preventing the application of garlic products in the treatment of diseases [18]. Therefore, it still remains a need to provide a standard procedure for the preparation of stable garlic-derived compositions which can be used in clinical or biological trials in order to objectively determine the properties attributable to this NP depending on their total or partial composition. 

To avoid most of the problems previously cited, we employed a thiosulfinate-enriched *Allium sativum* extract obtained by a standardized patented protocol from a purple garlic variety from Las Pedroñeras (Spain), the only European region with a protected geographical indication for garlic. We aim to validate, from both experimental and economic points of view, the use of a thiosulfinate-enriched *Allium sativum* extract as a source of active principles to pharmacological approaches. In particular, we aim to provide an evidence for the use of this new lyophilized garlic extract as a coadjuvant for chemotherapy drugs to increase their activity in treating colorectal tumors and to analyze the economic savings that a combined therapy could offer. 

## 2. Results

### 2.1. The Thiosulfinate-Enriched Allium sativum Extract Decreased Viability and Increased Cytotoxicity in Colon Cancer Cells

As mentioned in the Material and Methods section, we used allicin content to refer the dosage of the thiosulfinate-enriched *Allium sativum* extract that we employed in this work. The full list of organic and inorganic compounds contained in the garlic derivate used in this study is presented in Table 1.

**Figure 1:**
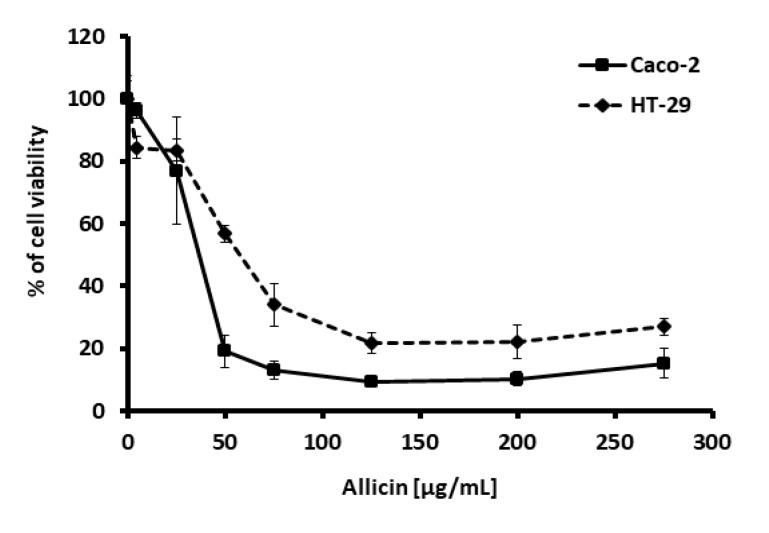
Effect of various thiosulfinate-enriched *Allium sativum* extract concentrations on cell viability of the colon cancer cell lines Caco-2 and HT-29, after 24 h incubation. Neutral red assays were performed, and the percentage of viable cells was calculated. Values represent means of six replicates ± SD of one representative experiment.

First, we tested the effect of the thiosulfinate-enriched *Allium sativum* extract on colon cancer cell viability. For this purpose, Caco-2 and HT-29 cells were exposed to increasing doses of the thiosulfinate-enriched *Allium sativum* extract, measured as increasing concentrations of allicin. A clear reduction in cell viability was observed with all the methods used. Neutral red assay revealed a marked decrease in cell viability (≥70%) in *Allium sativum* extract-treated cells (from 75 µg/mL allicin), after just 24 h of incubation (Figure 1). 

**Figure 2:**
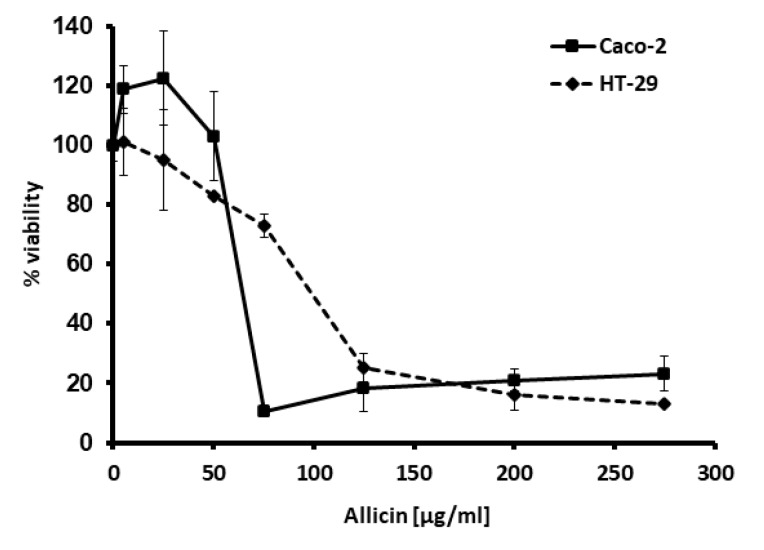
Effect of various thiosulfinate-enriched *Allium sativum* extract concentrations on cell viability of the colon cancer cell lines Caco-2 and HT-29, after 24 h incubation. MTT assays were performed, and the percentage of viable cells was calculated. Values represent means of six replicates ± SD of one representative experiment.

Similarly, MTT assays showed a 60–80% cell demise in response to the thiosulfinate-enriched *Allium sativum* extract (from 100 µg/mL allicin) (Figure 2), using the same dose-response approach. 

This effect on cell viability impairment, due to increasing concentrations of the thiosulfinate-enriched *Allium sativum* extract, was further confirmed by the morphological observation under the microscope of HT-29, as shown in Supplementary Figure S1. Thus, the incubation for 24 h with increasing concentrations of allicin (2.5; 5; 10; 25; 50; 75 and 100 µg/mL) elicited the appearance of rounded and detached cells, typical symptoms of affected cells in culture. In fact, the damage produced by the thiosulfinate-enriched *Allium sativum* extract was much more pronounced and according to what was observed in the previous tests of cellular viability with increasing concentrations of allicin. 

**Figure 3:**
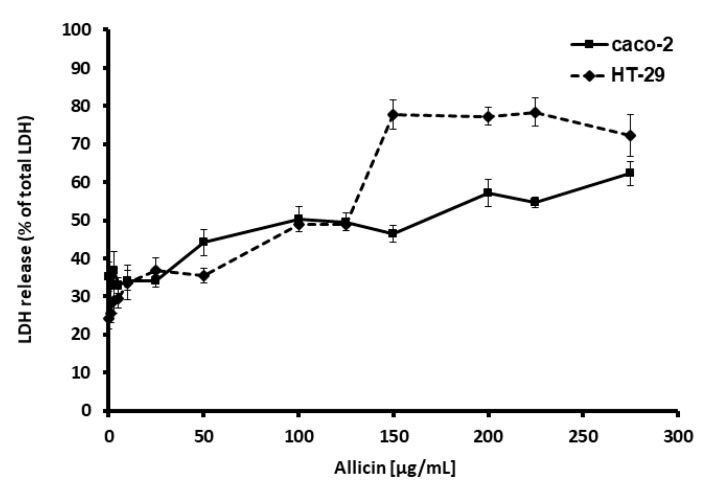
Cytotoxic effect of thiosulfinate-enriched *Allium sativum* extract on Caco-2 and HT-29 cells by the LDH test, after 24 h incubation. Concentrations are expressed as µg/mL of allicin. Values represent means of 6 replicates ± SD of one representative experiment.

Moreover, we further explored cell damage and cytotoxicity due to the thiosulfinate-enriched *Allium sativum* extract incubation with Caco-2 and HT-29. LDH release, a signal of cell membrane impairment, which occurs when cell integrity is compromised, was also detected in the supernatants after 24 h incubation with different concentrations of the thiosulfinate-enriched *Allium sativum* extract, as seen in Figure 3. All these results point out that the thiosulfinate-enriched *Allium sativum* extract can affect colon cancer cells survival in vitro and in a short period of time.

### 2.2. Analyses of the Type of Death Induced by the Thiosulfinate-Enriched Allium sativum Extract Treatment

In order to examine the type of cell death triggered by the garlic compound, we determined the amount of viable, apoptotic and necrotic cells after 24 h incubation with different thiosulfinate-enriched *Allium sativum* extract concentrations. We assessed apoptosis by the TUNEL method on HT-29 cells. As indicated in Table 2, we assessed a rapid decrease in cell viability, with increasing concentrations of the thiosulfinate-enriched *Allium sativum* extract (referred to as allicin), in terms of a higher percentage of early apoptotic cells. A significant increase in necrotic cells was not found. 

### 2.3. Combination of the Thiosulfinate-Enriched Allium sativum Extract with Chemotherapy Drugs

**Figure 4:**
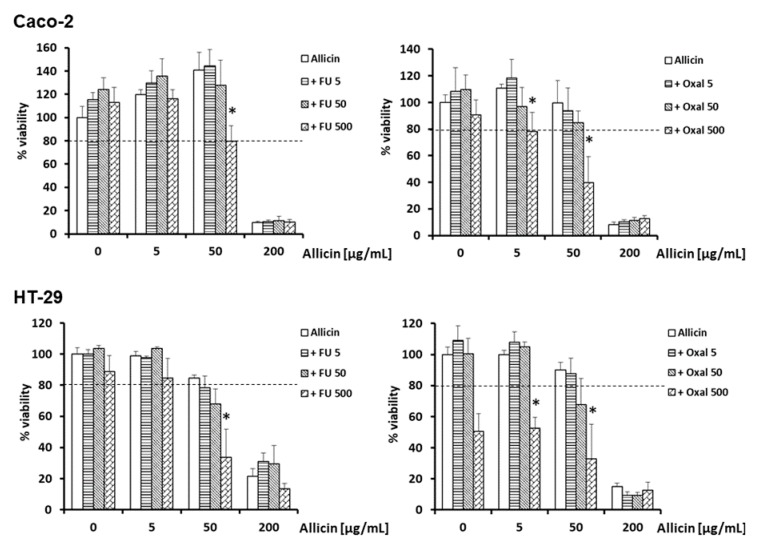
Effect of various *Allium sativum* extract concentrations (0, 5, 50 and 200 µg/mL; referred to allicin content, OX axis) with 5-FU or oxaliplatin (5, 50 and 500 µM) on cell viability of the colon cancer cell lines Caco-2 and HT-29, after 24h incubation. MTT assays were performed, and the percentage of viable cells was calculated. Values represent means of 6 replicates ± SD of one representative experiment. * *p* < 0.05 respect to its corresponding allicin or drug group.

After that, we explored whether the thiosulfinate-enriched *Allium sativum* extract could function as a coadjuvant in chemotherapy treatments, so we established a pattern of combined administrations (0, 5, 50 and 200 µg/mL; referred to allicin content), with three different concentrations of 5-fluorouracil (5-FU) or oxaliplatin (5, 50 and 500 µM). After 24 h incubation, the MTT assay revealed that, when combined with 500 µM 5-FU, 50 µg/mL of thiosulfinate-enriched *Allium sativum* extract enhanced the cell toxicity effects of 5-FU significantly, both in Caco-2 (−20%) and HT-29 (−66%). Moreover, 10 and 50 µg/mL of thiosulfinate-enriched *Allium sativum* extract plus 500 µM oxaliplatin were more potent at decreasing viability in Caco-2 (−22% and −60%, respectively) and HT-29 (−48% and −67%, respectively) cells, compared to 500 µM oxaliplatin alone (Figure 4). Results showed that combinations entailing the thiosulfinate-enriched *Allium sativum* extract and chemotherapy (5-FU/oxaliplatin) were able to reduce ≥ 20% cell viability (indicated by a dashed line in Figure 4) and were also statistically significant when compared to allicin or the chemotherapy drugs alone (indicated by an asterisk in Figure 4). This could be valuable to increase 5-FU/oxaliplatin efficacy or to avoid higher doses of 5-FU/oxaliplatin to get the same level of impairment.

### 2.4. Cost Effectiveness of Treatments Combining the Thiosulfinate-Enriched Allium sativum Extract with Chemotherapy

This part of the work aims to evaluate the costs associated to a chemotherapy treatment for both, standard oxaliplatin and alternative combination of oxaliplatin plus thiosulfinate-enriched *Allium sativum* extract and to determine the economic savings that could be derived from the use of combined therapy. This study was performed on the following basis: a standard patient (in terms of age and body weight) with advanced colorectal cancer undergoing 12 cycles of chemotherapy over 6 months, and receiving 85 mg/m^2^ of oxalipaltin in each cycle, according to actual treatment guides (National Comprehensive Cancer Network Guidelines for Treatment of Colon Cancer, 2019).

- Standard oxaliplatin dosage: 85 mg/m^2^ per cycle (equivalent to 1000 µM oxaliplatin)
- Patient dosing surface: 1.7 m^2^
- Number of chemotherapy cycles: 12
- Cost of commercial oxaliplatin vial (5 mg/mL, 20 mL): 132.26 € (including 4% VAT)

These data required approximately a total of 18 vials (5 mg/mL, 20 mL) ×/132.26 €, giving a cost of 2380.68 € for standard treatment.

The combined chemotherapy treatment was based on the following data, and calculated considering previous results for equivalent dosages that produced a similar cytotoxic effect (i.e., 500 µM oxaliplatin + 50 µg/mL allicin vs. 1000 µM oxaliplatin; data not shown):

- Combined oxaliplatin dosage: 42.5 mg/m2 per cycle (equivalent to 500 µM oxaliplatin)
- Combined thiosulfinate-enriched *Allium sativum* extract (Aliben©) dosage: 2.67 g per cycle (equivalent to 50 µg/mL allicin)
- Patient dosing surface: 1.7 m^2^
- Number of chemotherapy cycles: 12
- Cost of commercial oxaliplatin vial (5 mg/mL, 20 mL): 132.26 € (including 4% VAT)
- Cost of commercial Aliben©: 5 €/g, including 4% VAT (1 g of Aliben© contains 7.030 mg of allicin)

The combined treatment requires an approximate total of nine vials of oxaliplatin (5 mg/mL, 20 mL, 132.26 € each vial) and 32 g of Aliben© to achieve a dosage of 500 µM oxaliplatin + 50 µg/mL allicin in each cycle. This alternative treatment costs 1350.34 €. These results show that, on the same cellular impairment level, combined treatment might reduce the cost by 43.3%.

The total number of new invasive cancer cases in 2015 in Spain was 247,771 (148,827 in men and 98,944 in women), the most common being colon–rectum cancer, with 41,441 new cases [19]. Taking into account that 50% of colon cancer patients would be treated with chemotherapy, the combined alternative treatment using a thiosulfinate-enriched *Allium sativum* from Aliben© could provide 21.35 million € savings per year to the Public Spanish Health System.

## 3. Discussion

In this work, we presented a novel standardized and lyophilized garlic extract (thiosulfinate-enriched), suitable for pharmacological testing and with potent anticarcinogenic effects. Importantly, this extract was obtained by a patented protocol from a purple garlic variety, which is stable for more than 10 months when stored at 4 °C (WO 2008/102036 A1. Method for obtaining a freeze-dried, stable extract from plants of the *Allium* genus). This fact enables proper storage and handling over time, compared to other garlic extraction methods. Besides, we provided evidence for the combination of this new lyophilized garlic extract and 5-FU or oxaliplatin as a likely novel chemotherapy treatment for colon cancer cells. This regimen not only proved to be effective in affecting colorectal cancer cells, but also proved to reduce the chemotherapy drug dosage, so that a 43% reduction in the economic cost of the therapy could be achieved. 

We tested the cytotoxic effects of the thiosulfinate-enriched *Allium sativum* extract in two different human colon cancer cell lines, Caco-2 and HT-29, with similar results in both cell lines for three different cell viability assays (Neutral Red, MTT and LDH). The concentrations of allicin that were found to significantly inhibit the proliferation of colon cancer cells in this study (43–60 μg/mL allicin, equivalent to 260–360 µM) were similar to those reported in a previous study [20], but lower than others, where concentrations of 80 to 166 μg/mL [21] or even 480 μg/mL [22] were needed to achieve significant inhibition. A possible explanation for the differential efficacy of the garlic extract may be that the allicin contained in the thiosulfinate-enriched *Allium sativum* extract was obtained in situ (see Table 1) and not, as in the other studies, synthetically from its precursor. This has the advantage of a prolonged integrity of allicin and other thiosulfinates present in Aliben©.

Our data show that allicin is an inhibitor of cell viability and cell proliferation in a concentration dependent manner, something that has been well established to date by a number of works dealing with its anticarcinogenic and antitumorigenic capabilities [9,20,23,24,25,26]. However, the chemical properties of the lyophilized extract used in our work makes it suitable for different pharmacological approaches (i.e., water solution, hydroalcoholic lotion, hydrogel, cream).

Research has revealed that allicin targets multiple pathways implied in the inhibition of cancer development [27], including cell cycle arrest [21,28], the induction of apoptosis [29,30,31,32,33,34], the induction of histone acetylation [35] and the inhibition of angiogenesis [36]. Some of these studies dealing with the induction of apoptosis by allicin have also been reported for Caco-2 and HT-29 [20,34,37]. In our study, we also confirmed the induction of apoptosis in HT-29 cells incubated with the thiosulfinate-enriched *Allium sativum* extract for 24 h (see Table 2). 

Because allicin exhibits other pharmacological effects, such as cardiovascular and anti-microbial effects, this compound can be classified as a promiscuous agent [38]. However, if we considered promiscuity as versatility, it may be an advantage for cancer chemotherapeutic agents, because the pathogenesis of cancer is complex, involving abnormalities in multiple checkpoints and signaling pathways. As allicin targets multiple signal transduction pathways, it is also conceivable that this lyophilized garlic extract may prove useful in combination with mechanistically distinct chemotherapeutic agents, such as the ones that we propose. In fact, the use of natural compounds for combinatorial therapy in cancer treatment is a new promising line of research [39,40,41]. Moreover, because of its pharmacological safety, this garlic extract has been proposed to be used alone to prevent cancer and in combination with chemotherapy to treat cancer [42].

## 4. Materials and Methods

### 4.1. Thiosulfinate-Enriched Allium sativum Extract

The garlic extract was obtained following a standardized and patented protocol for the production of a new lyophilized garlic extract (WO 2008/102036 A1), commercially available under the brand name Aliben© (Aliben Foods S.L., Ciudad Real, Spain). The procedure was as follows: purple garlic was provided by Coopaman (Las Pedroñeras, Spain). The garlic was used with 1 month of maturation, and it was maintained at 4 °C from harvesting. The solvents used were ethanol (purity 96% *v/v*) and acetone (purity 99.5%). Other products were hydrochloric acid (purity 37%), methanol (HPLC-isocratic-preparative) for instrumental analysis and diallyl disulfide (purity 99%), used as an internal standard and provided by Panreac Quimica (Barcelona, Spain). Ethanol and acetone garlic extractions were obtained in a stirred tank extractor with the conditions optimized in a previous work, to maximize the yield of the process [43], according to the next procedure: extractions were performed by introducing 25 g of freshly milled garlic into a 1 L extractor along with 400 mL of the appropriate solvent (ethanol and acetone). The stirring system consisted of a jar-test Vittadini 6-P model Isco (Rome, Italy), with digital control of the stirring speed and set at 175 rpm. The extraction process was continued for 2 h, the mixture was filtered, and the solvent was evaporated using an R-114 rotary evaporator Büchi (Barcelona, Spain). The resulting thiosulfinates content was determined by HPLC analyses, using the procedure described below. 

### 4.2. HPLC Analyses of Allium sativum Extract

The thiosulfinate content of garlic was determined using a Shimadzu HPLC apparatus (Duisbrug, Germany), as outlined previously [43] using a methanol/water (50:50) mobile phase and a Supelcosil C18 (150 × 4.6 mm, i.d. = 5 μm) stationary phase provided by Analisis Vinicos S.A. (Tomelloso, Spain). Solute detection was accomplished by using an UV-vis detector, set at 254 nm. The mobile phase flow rate was 1 mL/min. Diallyl disulfide was used as internal standard. A typical composition of the compounds presented in the extract used in this study is shown in Table 1. We employed diallyl thiosulfinate (allicin) concentration as the reference for the elaboration of the experimental treatments.

### 4.3. Cell Culture and Chemotherapy Drugs

The human colorectal cancer cell lines Caco-2 and HT-29 were obtained from the American Type Culture Collection ([Caco2] ATCC^®^ HTB-37™ and [HT-29] ATCC^®^ HTB-38™). Cells were maintained in Dulbecco’s Modified Eagle Medium (DMEM, Sigma Aldrich, Darmstadt, Germany), supplemented with 10% fetal bovine serum and antibiotics (penicillin 100 IU/mL and streptomycin 100 μg/mL, Sigma Aldrich, Darmstadt, Germany). Cell cultures were kept at 37 °C in an atmosphere of 5% CO_2_. Chemotherapeutic drugs (5-FU and oxaliplatin) were obtained from Accord Healthcare S.L.U. (Barcelona, Spain).

### 4.4. Cell Proliferation—Neutral Red Uptake

The neutral red uptake assay provides a quantitative estimation of the number of viable cells in a culture. It is based on the ability of viable cells to incorporate and bind the supravital dye neutral red in the lysosomes. Caco-2 and HT-29 were seeded in 96-well tissue culture plates (1 × 10^4^ cells/well), and the day after, cells were incubated for 24 h with different thiosulfinate-enriched *Allium sativum* extract concentrations (allicin ranging from 1 to 275 μg/mL). The plates were then incubated for 2 h with DMEM medium containing 40 µg/mL neutral red (Sigma Aldrich, Darmstadt, Germany). The cells were subsequently washed with phosphate buffered saline and the retained dye was extracted with a solubilization solution (1% glacial acetic acid, 50% ethanol and 49% distilled water) and the absorbance was read using a spectrophotometer (Dynex Spectra MR, Chantilly, VA, USA) at 540 nm. 

### 4.5. Cell Proliferation—Mitochondrial Activity (MTT)

The (3-(4,5-Dimethylthiazol-2yl)-2,5-diphenyl-tetrazolium bromide) (MTT) assay estimates viable cells with active metabolism, that convert MTT into a purple colored formazan product. It was performed based on a previously described method, with slight modifications (20). Briefly, after exposure to the treatments (thiosulfinate-enriched *Allium sativum* extract and 5-FU/oxaliplatin), 100 µL of 0.5 µg/mL MTT (Sigma-Aldrich, Darmstadt, Germany) was added to each well and further incubated in the dark at 37 °C, 5% CO_2_. After 4 h, the medium was aspirated and 100 µL of DMSO added to dissolve the formazan product. Plates were placed on a shaking incubator for 15 min and read at 570 nm by a microplate reader (Dynex Spectra MR, Chantilly, VA, USA). Each treatment was tested in sextuplicate and the whole experiment was repeated twice. Data were expressed as a percentage of viable cells referred to control cells treated with vehicle (complete DMEM). 

### 4.6. Cytotoxicity Assay—Lactate Dehydrogenase (LDH) Release

CytoTox 96® Non-Radioactive Cytotoxicity Assay (Promega, Madison, WI, USA) measures lactate dehydrogenase (LDH), a stable cytosolic enzyme that is released upon cell lysis. It was only used to study the cytotoxicity of this thiosulfinate-enriched *Allium sativum* extract. Thus, 1 × 10^4^ cells/well were seeded in 96-well plates, and the day after incubated for 24 h with different extract concentrations (allicin ranging from 1 to 275 μg/mL). The activity of LDH was determined by the conversion of lactate to pyruvate and the subsequent reaction thereof with a tetrazolium salt to form formazan. Absorbances were measured with a spectrophotometer (Dynex Spectra MR, Chantilly, VA, USA) at 490 nm and were proportional to the number of damaged cells in the culture. Two independent experiments were performed with six replicates for each condition (cell type and concentration).

### 4.7. Morphology Analysis—Optical Microscopy Images

The effect of the thiosulfinate-enriched *Allium sativum* extract on cell morphology after 24 h of incubation was studied by optical microscopy. HT-29 morphology was imaged by optical microscopy using a Nikon ECLIPSE Ti-U microscope (Tokyo, Japan) at 10x, before the corresponding cell proliferation assay (Supplementary Figure S1).

### 4.8. Apoptosis Assay—TUNEL Method

The effect of different thiosulfinate-enriched *Allium sativum* extract concentrations on cell fate after 24 h incubation was studied by the ApopTag^®^ Fluorescein Direct In Situ Apoptosis Detection Kit (Sigma-Aldrich, Darmstadt, Germany), which detects apoptotic cells in situ by the direct TUNEL method. The functional state of HT-29 cells was analyzed by flow cytometry following the manufacturer’s instructions (Table 2). At least 5000 events were acquired in a FACScan cytometer (Becton Dickinson, Franklin Lakes, NJ, USA) and analyzed using the CellQuest™ Pro software (Becton Dickinson, Franklin Lakes, NJ, USA). 

### 4.9. Statistical Analysis

The GraphPad PRISM software 4.0 (San Diego, CA, USA) was used for the statistical analysis. Comparisons between two experimental groups were performed with Student *t*-tests. Statistical significance was considered at the 0.05 level.

## 5. Conclusions

Our results suggest that, as a consequence of the co-administration of thiosulfinate-enriched *Allium sativum* extract with lower doses of the chemotherapeutic agents, a similar anti-proliferative effect to that when chemotherapy drugs are administered alone could be achieved, at higher doses, so that a reduction in toxic side-effects could be elicited. Moreover, our financial analysis also highlights the potential economic savings of a combined treatment using the thiosulfinate-enriched *Allium sativum* extract as a coadjuvant for chemotherapy, pointing it out as a promising natural product and functional food for treating tumoral cells. 

## 6. Patents

Patent WO 2008/102036 A1. Method for obtaining a freeze-dried, stable extract from plants of the *Allium* genus.

National patent (Spanish Trademark number ES2675282A1). *Allium sativum* extract, its use for the manufacture of a medicinal product for the treatment of diseases, and its obtaining procedure.

## Figures and Tables

**Table 1:** Composition of the thiosulfinate-enriched *Allium sativum* extract. List of organic and inorganic compounds present in the lyophilized *Allium sativum* extract from Las Pedroñeras (Ciudad Real, Spain), under optimized conditions.

Compound	Concentration (µg/mg)	Compound	Concentration (µg/mg)
Allyl-1-propenyl thiosulfinate	31.023	K	3974.85
Dimethyl thiosulfinate	18.302	Si	3665.76
Dimethyl tetrasulfide	6.619	P	1188.87
**Diallyl thiosulfinate (allicin)**	5.617	Cu	298.16
Prostaglandin E1	4.831	Mg	188.41
Methyl allyl disulfide	4.725	Ca	159.12
Allylmethyl + Methyl-allyl thiosulfinate	4.581	Na	102.41
Methyl allyl sulphide	3.575	Fe	95.84
Propyl-methyl + Methyl-propyl thiosulfinate	3.394	B	89.45
Vitamin E (α-tocopherol)	3.072	Cr	26.37
1-propenyl-allyl + allyl-propyl thiosulfinate	1.760	Zn	10.60
Di-propyl thiosulfinate	1.653	Cd	9.48
Di-methyl pentasulfide	1.631	Se	9.37
Propyl-allyl thiosulfinate	1.591	Mn	1.18
Di-allyl trisulfide	0.736	Co, Hg	Non Detected
Inulin	0.103	Al	Non Detected
(E,Z)-Ajoene	0.068	Ni	Non Detected

**Table 2:** Analysis of type of death. Percentage of viable, apoptotic (early and late) and necrotic cells after 24 h incubation, with increasing concentrations of the thiosulfinate-enriched Allium sativum extract (10, 50 or 100 µg/mL allicin).

	Control	Allicin [10 µg/mL]	Allicin [50 µg/mL]	Allicin [100 µg/mL]
**Viable cells**	98.40%	94.20%	74.50%	54.12%
**Early apoptotic cells**	0.31%	4.53%	22.24%	44.04%
**Late apoptotic cells**	0.16%	0.16%	0.26%	1.43%
**Necrotic cells**	1.09%	1.11%	3%	2.41%

## Supplementary Materials

Supplementary Materials can be found at https://www.mdpi.com/1422-0067/21/8/2766/s1.

## Abbreviations

5-FU	5-fluorouracil
MTT	3-(4,5-Dimethylthiazol-2-yl)-2,5-Diphenyltetrazolium bromide
LDH	lactate dehydrogenase
NP	natural products
HPLC	high-performance liquid chromatography
UV-vis	ultraviolet-visible
DMEM	Dulbecco’s modified Eagle medium
VAT	value added tax
