# Supplementary material for: Cost Effective Use of a Thiosulfinate-Enriched Allium sativum Extract in Combination with Chemotherapy in Colon Cancer
Source: Int J Mol Sci. 2020 Apr 16;21(8):2766. doi: 10.3390/ijms21082766 (PMC7216288; doi:10.3390/ijms21082766)
Supplement: Supplementary file 1 [file ijms-21-02766-s001.pdf]

Supplementary Figure 1.

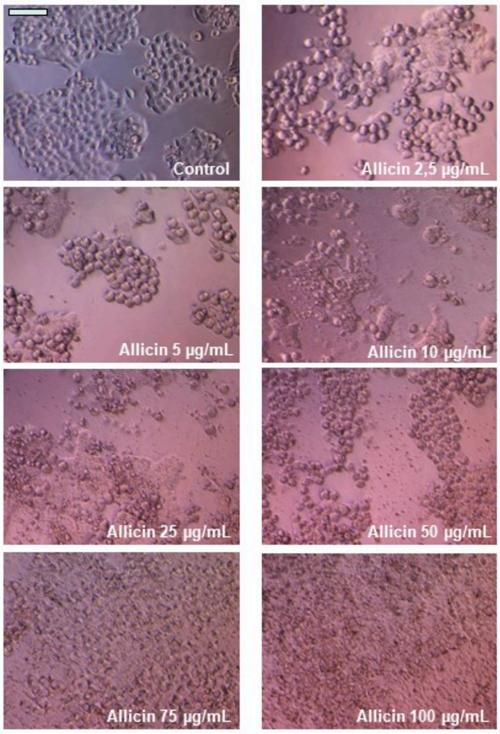

We show the cell morphology of HT-29 cells after 24 h incubation with different *Allium sativum* extract concentrations (referred to allicin content). In detail, 2.5 x 10^5 cells were seeded in 6-well plates, and each well was incubated during 24 h with different *Allium sativum* extract concentrations (0, 2.5, 5, 10, 25, 50, 75 and 100 µg/mL; referred to allicin content). One representative image is shown for each condition.
Scale bar = 100 µm.
